# Supplementary figures and images for: Molecular characterization of circulating Salmonella Typhi strains in an urban informal settlement in Kenya
Source: PLoS Negl Trop Dis. 2022 Aug 25;16(8):e0010704. doi: 10.1371/journal.pntd.0010704 (PMC9451065; doi:10.1371/journal.pntd.0010704)

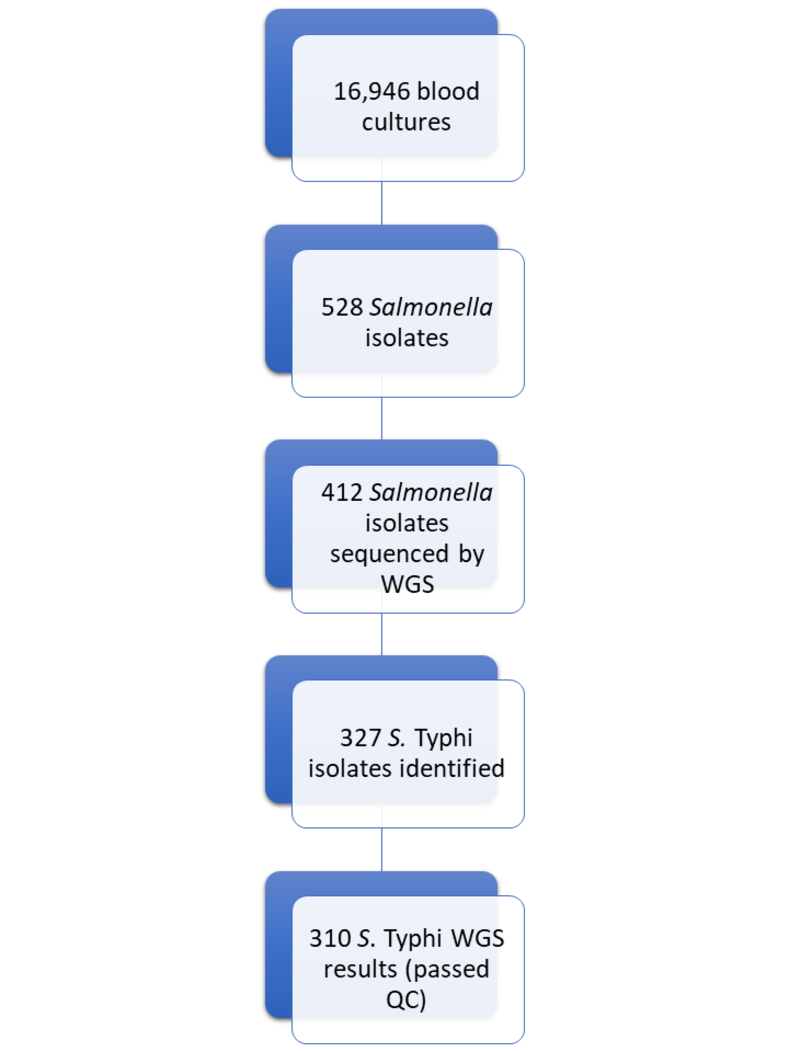

Supplement: S1 Fig — (TIF) [file pntd.0010704.s001.tif]
