# Supplementary material for: Molecular characterization of circulating Salmonella Typhi strains in an urban informal settlement in Kenya
Source: PLoS Negl Trop Dis. 2022 Aug 25;16(8):e0010704. doi: 10.1371/journal.pntd.0010704 (PMC9451065; doi:10.1371/journal.pntd.0010704)
Supplement: Attachment — Submitted filename: Responses to Reviewer Comments - Ochieng et al. 2022.docx [file pntd.0010704.s004.docx]

We thank the reviewers for their thoughtful comments. We’ve carefully considered the suggestions and offer the responses and amendments highlighted in yellow for your consideration. A revised copy of the manuscript with comments tracked is attached.

- Why authors used the ggtree package if it has the same function like ITOL for the annotation of phylogenomic trees.

Both packages are useful for the visualization of phylogenetic trees but have slightly different features for doing so. We used iTOL initially to visualize the cgMLST tree however, when we completed the time tree we found that we were not able to visualize certain node attributes of the time tree using the version of iTOL available at the time, so we used ggtree to visualize the molecular clock for this purpose.

-As mentioned  in Paragraph (lines 262-266), three different plasmid markers were detected among isolates; the authors have not determined if any of the resistance genes detected are linked to a specific plasmid replicon type, if so, it is important to reconstruct the plasmid sequences from whole genome sequences.

Thank you for this suggestion. We agree that reconstruction of plasmid sequences is an important aspect here. This study was conducted using short-read sequencing, and reconstruction of plasmid sequencing is challenging with short read data due to IS elements and other repeat elements in the plasmid sequence. Further *Salmonella* plasmids can harbor multiple replicons on a single plasmid, or harbor multiple plasmids with different replications, which is challenging to distinguish with this type of data. Further study is under way to long read sequence a subset of isolates from this study for a more thorough picture of the plasmids identified in this study and to facilitate comparison to known typhoid plasmid sequences. This work is planned to be published as part of a separate study, but we updated the manuscript’s limitations section as detailed below to include this as a limitation in the study.

“This study utilizes short-read sequencing which provides valuable information to perform genomic characterization, however complete assembly of plasmids is challenging with this technology due to insertion sequence elements and other repeat elements in the plasmid sequence. Additional study is required to associate specific resistance genes with specific plasmids, as well as facilitate comparison with known reference plasmids previously identified in *S.* Typhi.”
